# Tracheobronchial ossification in children: a case report and review of the literature

**DOI:** 10.3389/fped.2025.1552947

**Published:** 2025-05-22

**Authors:** Yan Ruan, Shan Chen, Ling Zhang, Huiyun Huang, Weiwei Tang, Lizhi Li

**Affiliations:** ^1^ Department of Pediatrics, Fuzhou Pulmonary Hospital of Fujian, Fuzhou, Fujian, China; ^2^ Department of Laboratory, Fuzhou Second General Hospital, Fuzhou, Fujian, China; ^3^ Department of Pediatric Surgery, Provincial Clinical Medical College, Fujian Medical University, Fuzhou, Fujian, China

**Keywords:** pediatrics, tracheobronchial, tracheobronchial ossification, bronchoscopy, case report

## Abstract

**Objective:**

This study aimed to report a case of tracheobronchial ossification (TO) in a child and review the related literature on its clinical signs, diagnosis, differential diagnosis, pathological characteristics, points, and treatment measures.

**Methods:**

A retrospective analysis was conducted on the clinical data of one pediatric patient with TO admitted to the Department of Pediatrics, Fuzhou Pulmonary Hospital. In addition, three relevant articles published up to July 2024 were reviewed from both domestic and international databases, including PubMed, China National Knowledge Infrastructure (CNKI), Wanfang Data, Chinese Biomedical Literature Database (CBM), Web of Science, and the Cochrane Library. The clinical features of a total of four pediatric cases of tracheobronchial osteochondroplasia were summarized and analyzed.

**Results:**

A 12-year-old girl with a 5-year history of symptoms was retrospectively reviewed. The patient presented with chronic cough, sputum, hemoptysis, and exertional dyspnea. Comorbidities such as bronchiectasis with cavitation, pneumonia, *Klebsiella pneumoniae* infection, and sinusitis were observed. Imaging revealed airway wall nodules and structural lung changes. Bronchoscopy showed scattered white nodules; histopathology confirmed mucosal ossification with focal bone marrow formation. Symptomatic improvement was achieved after anti-infective and supportive treatment. A 4-year follow-up revealed recurrent pneumonia and progression of bronchiectasis. Three additional pediatric TO cases (aged 6 months to 9 years) were identified in the literature. Presentations included respiratory symptoms and recurrent infections. Pulmonary function abnormalities, typical radiological and bronchoscopic features, and histopathological evidence were variably reported. Treatments ranged from symptomatic therapy to bronchoscopic intervention. One patient died, one remained stable, and follow-up data were limited.

**Conclusion:**

TO can develop in children, with atypical chest computed tomography and bronchoscopic changes. Histopathological examination can confirm the diagnosis, and symptomatic treatment is the mainstay.

## Introduction

Tracheobronchial ossification (TO) is a rare benign airway disease characterized by multiple submucosal cartilaginous and bony nodules in the trachea and bronchi that protrude into the lumen, leading to wall thickening, lumen deformation, and stenosis as the disease progresses. The case of a patient with TO admitted to our hospital in November 2020 is summarized as follows.

## Case presentation

### Chief complaint and admission history

A 12-year-old girl was admitted to the hospital with “recurrent cough and yellow pus sputum for more than 5 years, aggravated for 1 week.” Her symptoms of cough and yellow sputum had persisted for more than 5 years and were managed with treatment at a local hospital, resulting in temporary improvement. However, the symptoms frequently recurred. One week prior to admission, she experienced a recurrence of cough and yellow sputum, accompanied by hemoptysis. A tuberculin skin test [purified protein derivative (PPD)] was positive.

A chest x-ray scan revealed patchy, flocculent opacities in the upper and middle zones of the right lung and the upper zone of the left lung. Chest computed tomography (CT) demonstrated patchy, nodular, and flocculent hyperdense areas in all the lobes of the right lung and the upper lobe of the left lung. Cavitary lesions were observed in the upper and lower lobes of the right lung, along with evidence of bronchiectasis. Based on these findings, the initial consideration was “pulmonary tuberculosis to be ruled out.”

### Past medical and family history

The patient's birth history, past medical history, and family history were unremarkable.

### Physical examination

On physical examination, her vital signs were stable: respiration rate of 20 breaths/min, heart rate of 92 beats/min, weight of 56 kg, and peripheral oxygen saturation (SpO_2_) of 99%. Auscultation revealed coarse breath sounds bilaterally with a small amount of wet rales. There were no abnormalities on cardiac or abdominal examination.

### Bronchoscopic and histopathological findings

**Figure 1:**
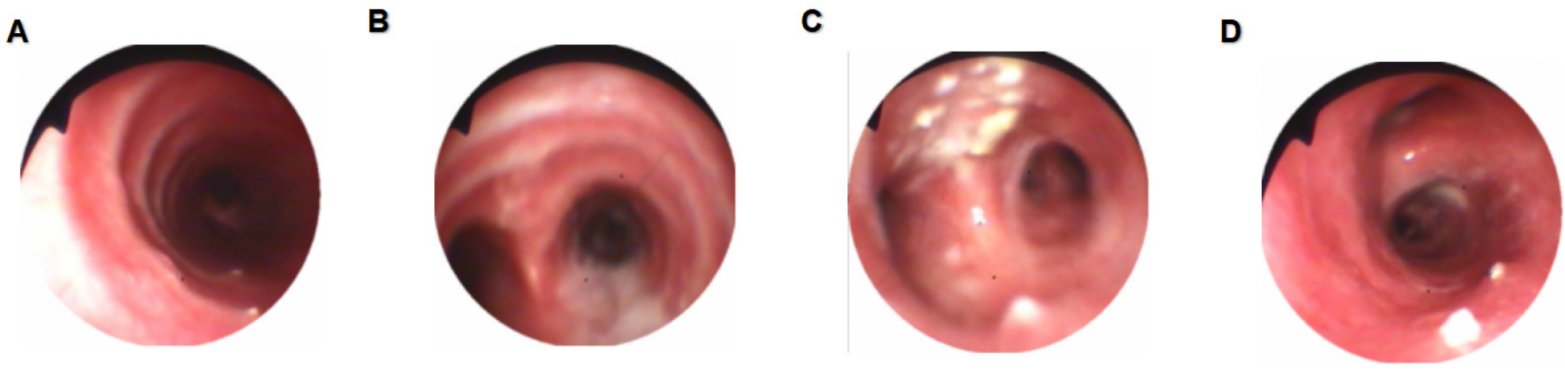
Bronchoscopic findings. **(A)** Scattered white nodules were observed in the trachea with clearly visible cartilage rings. **(B)** A large amount of purulent secretions was observed at the carina. **(C)** Marked mucosal hyperplasia with multiple white nodule-like lesions on the surface of the left upper bronchus. **(D)** A white nodule was observed in the right main bronchus.

**Figure 2:**
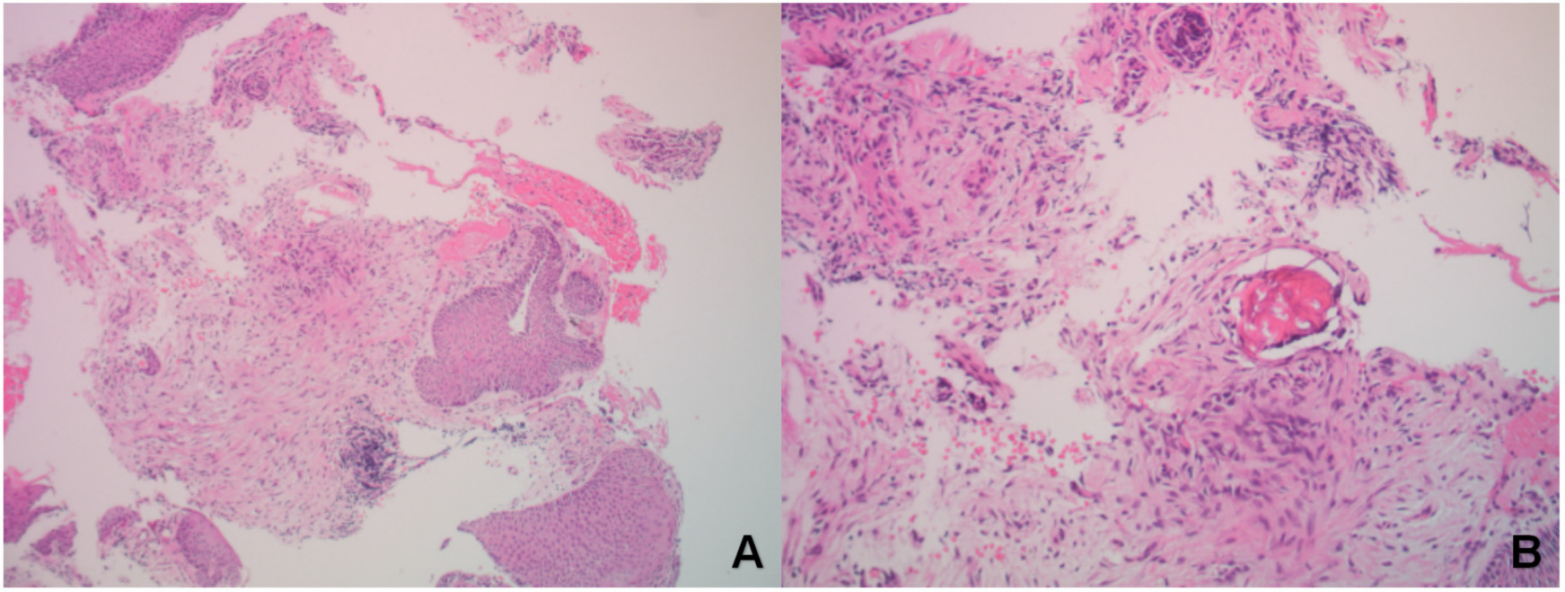
Histopathological findings. The bronchial mucosal epithelium showed hyperplasia with ossification and excessive keratinization. Stromal fibrous tissue hyperplasia and focal osteoid matrix formation were observed, consistent with a diagnosis of TO. **(A)** Hematoxylin and eosin (H&E) staining, magnification ×100. **(B)** H&E staining, magnification ×400.

Bronchoscopy revealed a patent tracheal lumen with mucosal congestion, edema, and purulent secretions. After suction, scattered small white nodules were observed, with clear cartilage rings visible. The lumens of the bilateral lobar and segmental bronchi were patent, showing mucosal congestion, edema, rough surfaces, multiple white nodules, and copious purulent secretions. The mucosa of the left upper lobe spur showed marked hyperplasia with multiple white nodular changes on the surface, which were resistant to suction (Figure 1). A biopsy of the mucosa of the left upper lobe revealed hyperplastic bronchial mucosal epithelium with ossification and keratosis, stromal fibrous tissue hyperplasia, and focal osteoid matrix formation, consistent with tracheobronchial osteochondrosis (Figure 2).

### Radiological findings

A chest x-ray scan showed that patchy flocculent shadows were visible in the middle and upper zones of the right lung and the upper zone of the left lung. A chest CT scan revealed multiple speckled, patchy, nodular, and cord-like hyperdense areas, along with ground-glass opacities in both lungs. Some lesions displayed blurred edges and pleural adhesions. Bronchiectasis was observed in some lesions, while other lesions exhibited translucent areas connected to the bronchi. Thickened bronchial walls were noted in the upper lobe of the right lung and part of the lower lobe of the left lung. A sinus CT scan revealed bilateral inflammation in the maxillary, ethmoidal, sphenoidal, and frontal sinuses.

### Microbiological and immunological workup

Cultures of sputum and bronchoalveolar lavage fluid identified *Klebsiella pneumoniae* (*odorata* subspecies).

The tuberculin skin test (PPD) measured 18 mm × 14 mm. The T lymphocyte spot (T-SPOT) test for tuberculosis infection was negative. Sputum tests for tuberculosis (smear, *Mycobacterium tuberculosis* drug-resistant locus detection, and culture) were negative. Similarly, tests on bronchoalveolar lavage fluid (RNA, DNA, drug-resistant locus detection, and culture for *M. tuberculosis*) were negative.

### Clinical course and management

The patient was diagnosed with TO, bronchiectasis with cavitation, *K. pneumoniae* pneumonia, and bilateral sinusitis. Symptomatic treatment, including anti-infective therapy, expectorants, and bronchoscopic suctioning, was administered, leading to symptom improvement and discharge.

**Figure 3:**
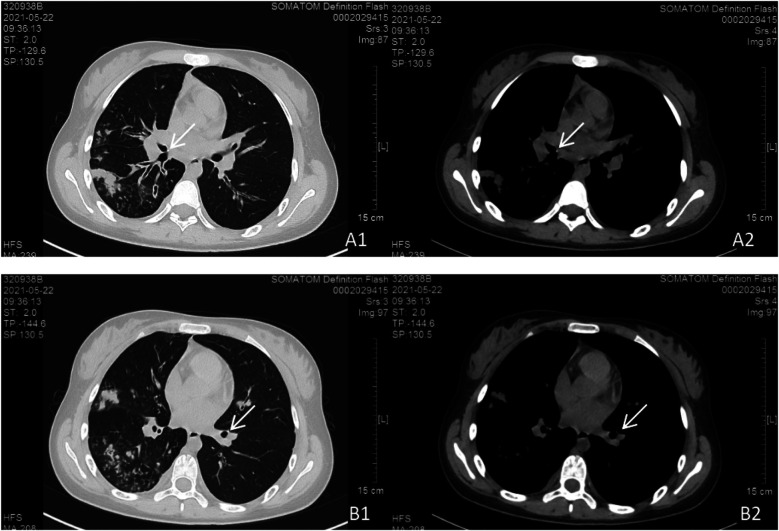
Chest CT scan findings. Bilateral pulmonary lesions were observed, with more extensive involvement in the right lung. **(A1, A2)** Parietal nodules adjacent to the right middle bronchus. **(B1, B2)** Parietal nodules adjacent to the basal segment of the left lower lobe.

However, 5 months later, the patient was rehospitalized due to cough and purulent yellow sputum. Chest CT (22 May 2021) showed resolution of the ground-glass opacity in the left lower lobe compared to the previous scan (20 November 2020), but there was an increase in the bilateral pulmonary lesions and air lucencies. Bronchial wall thickening persisted in the right upper and left lower lobes, with new bilateral pleural effusions (Figure 3). Bronchoscopy (24 May 2021) revealed a patent tracheal lumen with congested and edematous mucosa, mucopurulent secretions, and scattered white nodules with visible cartilage rings. Segmental bronchi also showed mucosal hyperemia, edema, roughness, and numerous purulent secretions. Biopsy was not performed due to inadequate tissue. Sputum culture identified *K. pneumoniae* (*ozenae* subspecies).

**Figure 4:**
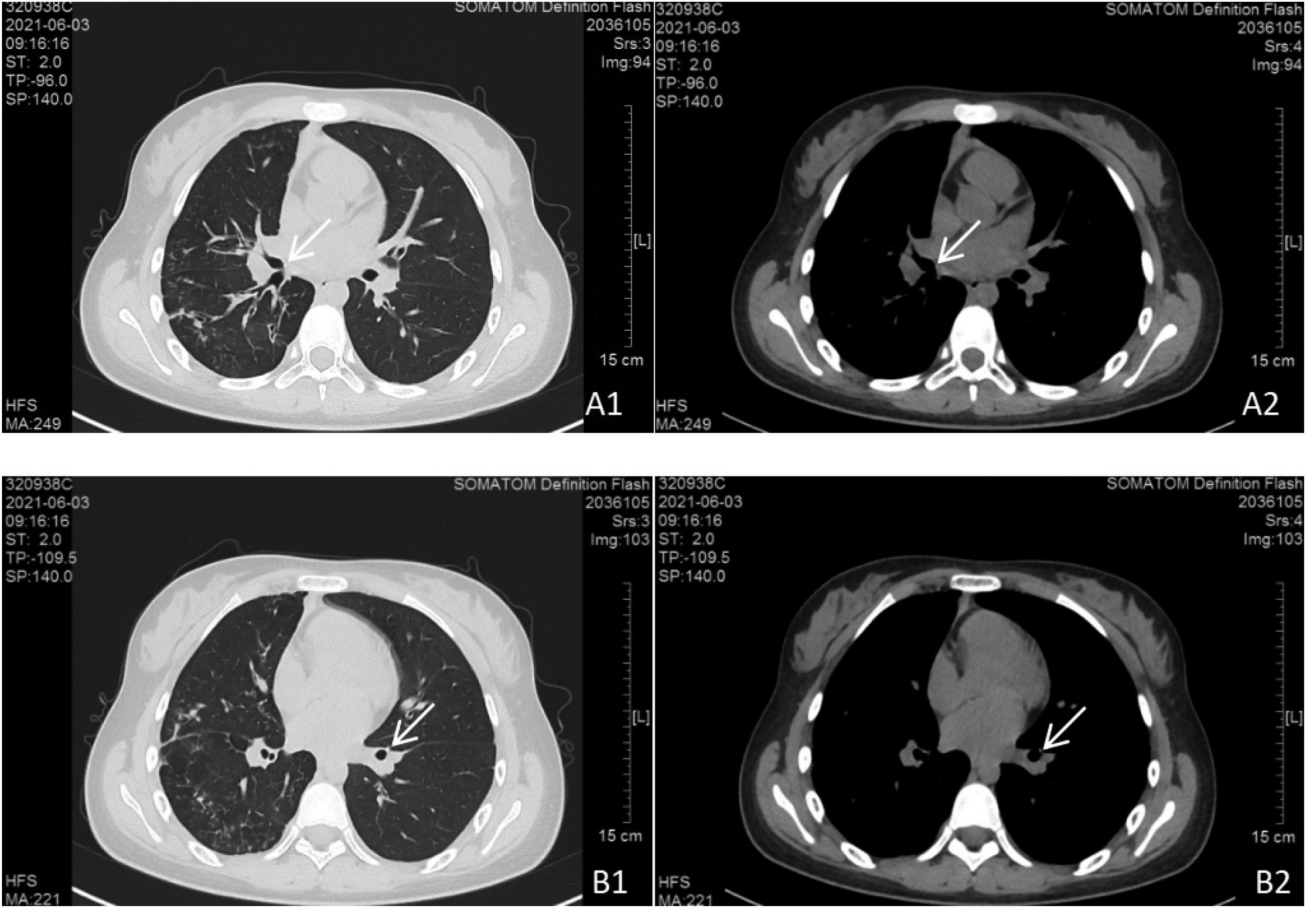
Follow-up chest CT scan. Bilateral pulmonary lesions showed significant absorption compared to Figure 3. **(A1, A2)** Persistent parietal nodules adjacent to the right middle bronchus. **(B1, B2)** Persistent parietal nodules adjacent to the basal segment of the left lower lobe.

The diagnosis remained unchanged. The same treatment regimen was applied, and follow-up CT (3 June 2021) demonstrated partial resolution of the lesions, shrinkage and closure of cavitations, persistent bronchial wall thickening, and absorption of pleural effusion (Figure 4). The patient was discharged after clinical improvement.

During the subsequent 4 years, the patient experienced recurrent episodes of pneumonia, all of which responded to empirical anti-infective treatment.

**Figure 5:**
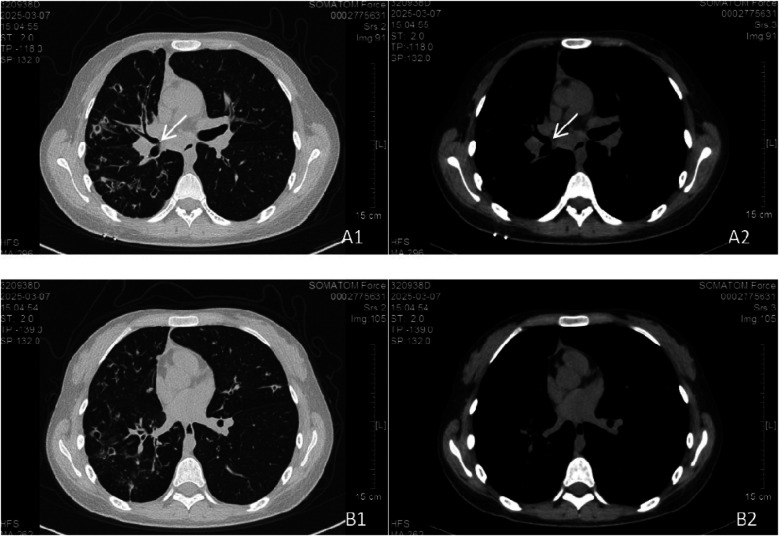
Chest CT scan at follow-up. The bilateral pulmonary lesions had progressed compared to Figure 4. Bronchiectasis with cavitation in the right lung increased in both number and size. Lesions in the left lung also increased. **(A1, A2)** Persistent parietal nodules adjacent to the right middle bronchus. **(B1, B2)** Parietal nodules in the basal segment of the left lower lobe were not visualized, possibly due to CT slice thickness or scan level.

**Figure 6:**
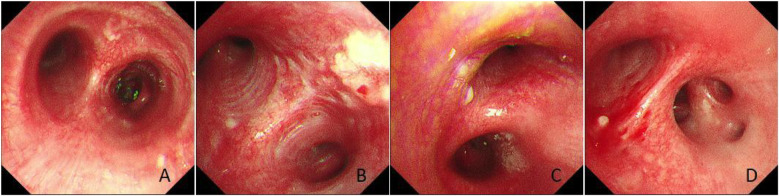
Bronchoscopic findings. **(A)** Scattered white nodules were observed in the trachea. **(B)** Nodule-like lesions were observed in the left upper bronchus. **(C)** A white nodule was observed in the right main bronchus. **(D)** A white nodule was observed in the right middle and lower bronchi.

**Figure 7:**
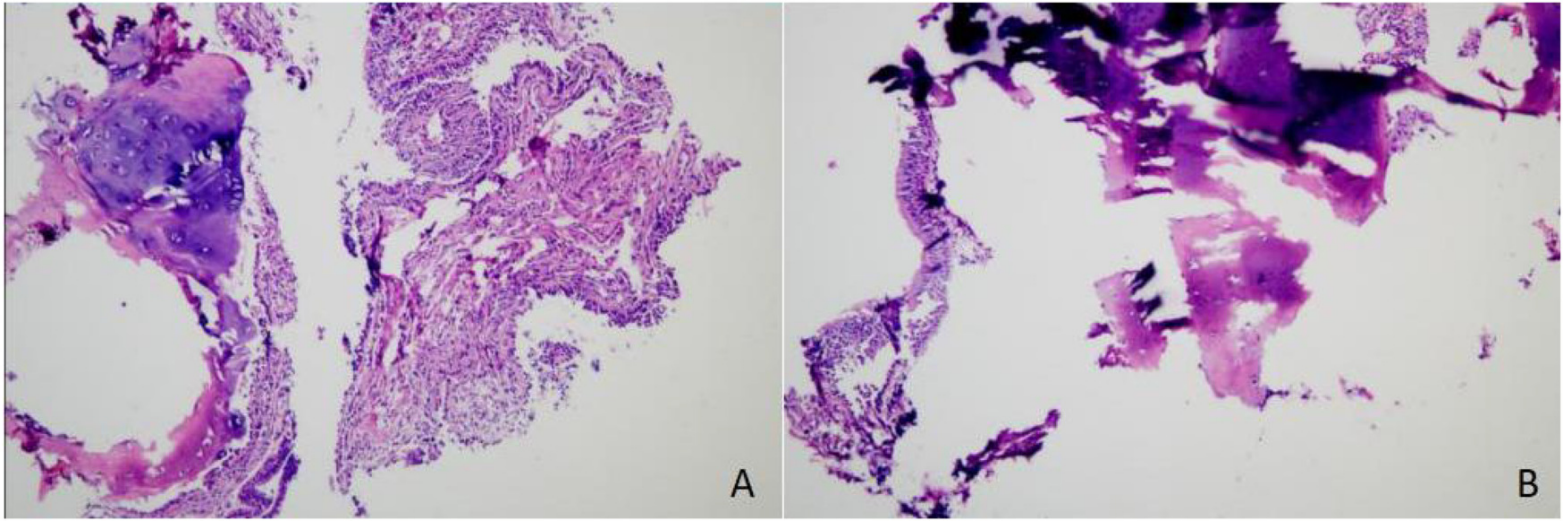
Histopathological findings. Chronic inflammation of the bronchial mucosa was observed, with interstitial ossification and focal formation of bone marrow cavities containing hematopoietic cells, consistent with a diagnosis of tracheobronchial osteochondroplasia. **(A)** Hematoxylin and eosin (H&E) staining, magnification ×100. **(B)** H&E staining, magnification ×400.

In March 2025, the patient was readmitted with cough, sputum production, and exertional dyspnea. Chest CT (7 March 2025) showed a progression of the bilateral pulmonary lesions, increased bronchiectasis and cavitation, and persistent bronchial wall thickening compared to 3 June 2021 (Figure 5). Bronchoscopy (10 March 2025) revealed scattered white nodules in the trachea, secretions in the segmental bronchi, and white nodule-like lesions in the left upper lobe and right middle lobe (Figure 6). A biopsy of the right middle lobe mucosa revealed chronic bronchial inflammation with interstitial ossification and focal bone marrow formation (Figure 7). Sputum culture again identified *K. pneumoniae* (*ozenae* subspecies).

The diagnosis was reaffirmed, and treatment with antibiotics, expectorants, and bronchoscopic suctioning led to symptomatic improvement and discharge.

### Literature search strategy

**Figure 8:**
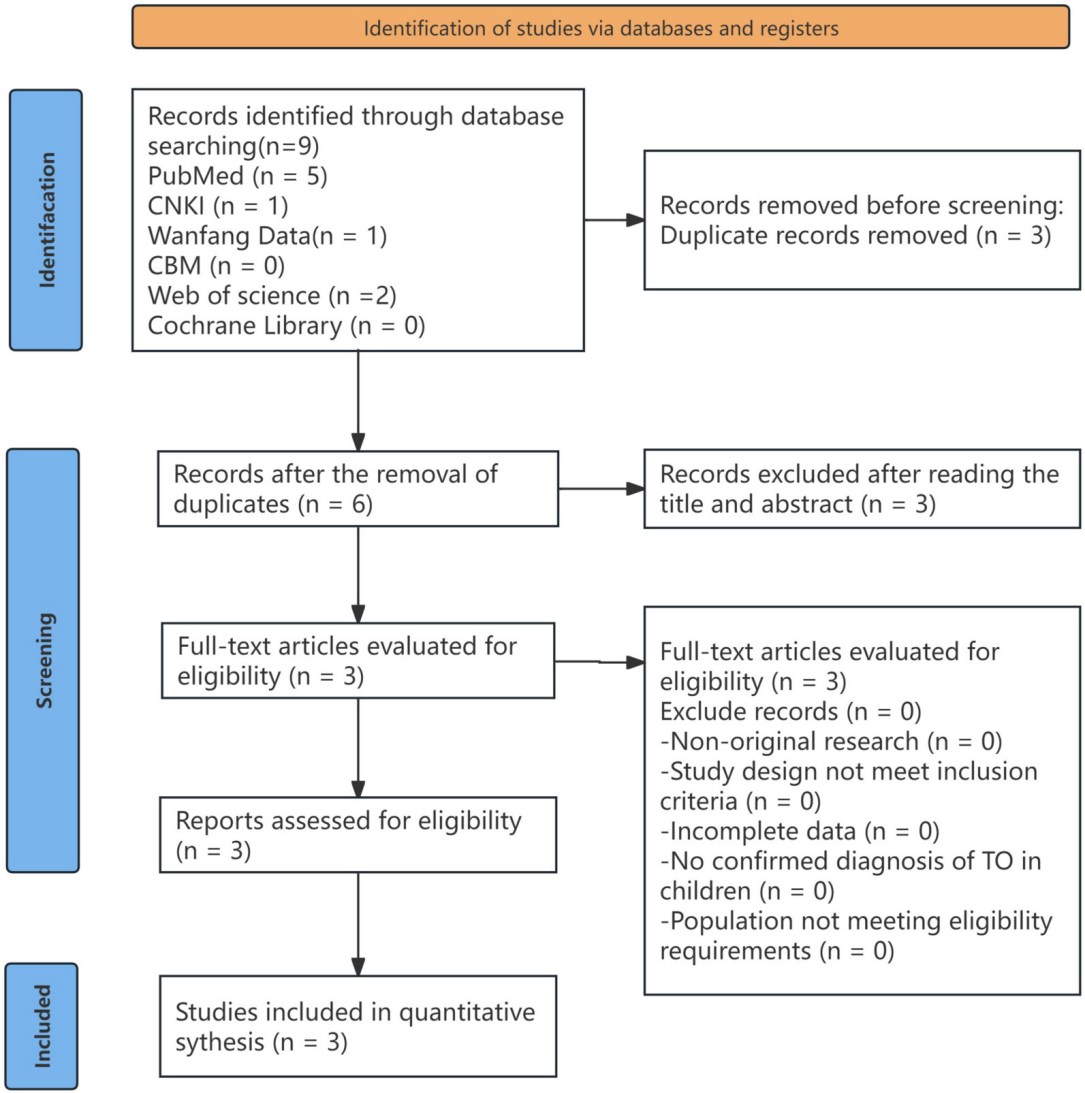
Flowchart of literature screening and selection of studies on TO in children.

A comprehensive literature search was conducted using the following databases: PubMed, CNKI, Wanfang Data, CBM, Web of Science, and the Cochrane Library. The keywords used included “tracheobronchial ossification,” “children,” “tracheobronchopathia osteochondroplastica, TO,” and “children.” The search was limited to articles published up to July 2024. There were no language restrictions. In addition, related articles recommended by each database were manually screened. Reference lists and citations of potentially relevant articles were also reviewed to identify additional eligible publications (Figure 8).

### Eligibility criteria

The inclusion criteria for literature selection were based on study design, participants, interventions, comparisons, and outcome measures. First, only randomized controlled trials (RCTs), non-randomized observational studies, and case reports were included. Reviews and studies with incomplete data were excluded. Second, the diagnosis of TO in children had to be explicitly confirmed. Two independent reviewers screened the retrieved literature for eligibility, and any discrepancies regarding inclusion or exclusion were resolved through discussion.

### Quality assessment

After inclusion, the data from the selected studies were extracted by a third reviewer. Information was summarized in a predesigned data extraction form, including the first author, year of publication, number of cases, age, symptoms, clinical signs, comorbidities, treatment, and follow-up. Information related to incomplete outcome data, selective reporting, and other potential sources of bias was also collected. The methodological quality of the included studies was assessed based on the criteria recommended by the Cochrane Collaboration, focusing on potential biases in study selection, performance, detection, attrition, and reporting.

## Results

### Diagnosis, treatment, and follow-up of the present case

#### General information

The patient was a 12-year-old girl with a 5-year history of symptoms.

#### Clinical presentation

She presented with chronic cough, sputum production, hemoptysis, and exertional dyspnea.

#### Complications

Bronchiectasis with cavitation, *K. pneumoniae* pneumonia, and bilateral sinusitis were identified.

#### Auxiliary examinations

Pulmonary function tests were within normal limits. A chest x-ray scan revealed radiographic features consistent with pneumonia and bronchiectasis with cavitation. Chest CT showed segmental bronchial wall thickening and changes related to the above complications. Bronchoscopy demonstrated scattered white nodules in the trachea and bilateral bronchi. A histopathological examination revealed chronic inflammation of the bronchial mucosa, interstitial ossification, and focal bone marrow cavity formation with hematopoietic cells.

#### Treatment and outcome

Symptomatic treatment, including antibiotics, antitussives, and expectorants, led to significant improvement in symptoms.

#### Follow-up

The patient was followed for over 4 years. Her condition remained generally stable, with intermittent episodes of pneumonia that responded well to anti-infective therapy. Follow-up chest CT revealed bilateral pulmonary lesions with progressive bronchiectasis and cavitation; some lesions had increased while others showed partial resolution. Cavities had increased in number and size compared to previous scans. Bronchial wall thickening persisted, and no significant changes were observed in the parietal nodules along the trachea and bronchi.

### Results of literature analysis

#### General information

Three cases were reported, including 1 boy and 2 girls, aged 6 months to 9 years. The duration of illness ranged from 6 months to 3 years.

#### Clinical manifestations

The patients presented with coughing in two cases, and dyspnea, weak cry, and cyanosis in one case.

#### Complications

The comorbidities were recurrent respiratory infections in two cases and pulmonary atelectasis in one case.

#### Auxiliary examinations

(1) Pulmonary function tests were conducted in all three cases, with results showing normal findings in one case, obstructive ventilation dysfunction in one case, and restrictive ventilation dysfunction in one case. (2) Chest x-ray scans were performed in one case, revealing irregular tracheal changes. (3) Chest CT scans were conducted in two cases, both showing typical imaging findings of TO. (4) Bronchoscopy was completed in all three cases, revealing characteristic TO findings. (5) Pathology was performed in two cases, with one case confirming TO histopathology and one case showing non-specific chronic inflammation (Table 1).

**Table 1:** Clinical data of the case reports from the literature.

Author	Year	Onset age	Gender	Disease duration	Symptoms	Pulmonary function	Chest x-ray scan	Chest CT scan	Bronchoscopy findings	Pathological findings
Ma et al. (1)	2014	6 months	Male	6 months	Inspiratory dyspnea with weak crying and paroxysmal cyanosis	Restrictive ventilatory dysfunction	None	Extensive ossification of the laryngeal, tracheal, and bronchial walls with stenosis of the upper trachea	Stenosis of the funnel-shaped region and upper trachea, with thickened mucosa and prominent longitudinal folds at the stenotic sites	None
Sant'Anna et al. (2)	2012	7 years	Female	2 years	Chronic cough	Normal	None	None	Extensive nodular mucosal irregularities extending from the trachea to the bronchi, with abnormal mucosal morphology	Non-specific chronic inflammatory process
Simsek et al. (3)	2006	9 years	Female	3 years	Chronic cough	Mild obstructive ventilatory dysfunction	Irregular trachea	Submucosal calcification in the trachea and main bronchi, with the posterior wall unaffected	Tracheal wall deformity and white nodular lesions on the anterior tracheal wall, with the lesions extending to both bronchi, forming oval structures on the mucosa	Submucosal osseous tissue observed beneath the epithelium of the nodules

#### Treatment and outcome

Symptomatic treatment (anti-infective therapy, cough suppression, and mucolytics) resulted in symptom improvement in two cases. One patient underwent bronchoscopic interventions, including balloon dilation, electrocautery, and cryotherapy, with significant improvement.

#### Follow-up

No relevant follow-up information was available for one child, one child died after 15 weeks of follow-up due to severe respiratory distress, one child had no significant progression of symptoms at 4 years of follow-up, and none of the children had follow-up chest CT or bronchoscopy findings.

## Discussion

TO is a chronic progressive benign disease characterized by multiple submucosal bony and cartilaginous nodular hyperplasia in the tracheobronchial tubes that protrude into the lumen. It is a rare disease, most often occurring in adults, with even rarer onset in children, and there are reports in the literature of TO in children at the ages of 6 months (1), 5 years (2), and 9 years (3), while the case reported here involves a 12-year-old patient.

The etiology and pathogenesis of TO remain unclear. Factors such as inflammatory stimuli, chemical or mechanical irritants, congenital abnormalities, degenerative changes, metabolic diseases, and amyloidosis may contribute to its development. Tajima et al. found that the interaction between BMP-2 and TGF-β1 was associated with the formation of calcified foci of TO (4). Hong et al. reported that tracheobronchial basal stem cells mediate ectopic bone and cartilage formation, and their dysfunction is associated with TO development (5). Hong et al. reported that tracheobronchial stem cells could mediate ectopic osteochondral formation, and their dysfunction was associated with the development of TO, while inflammatory features detected in tracheobronchial basal stem cells confirmed the hypothesis that chronic inflammation plays a role in the development of TO (5). The patients in the reports by Sant'Anna et al. (2) and Simsek et al. (3) and the child with TO reported in this article had a history of chronic cough and recurrent respiratory tract infections. It is hypothesized that the occurrence of TO in children may also be associated with chronic inflammation, but the causal relationship still needs further study. Ma et al. (1) reported that children with TO presented with dyspnea immediately after birth, and their birth history and family history were not specific, so we hypothesize that the etiology may be related to congenital factors. However, this lacks definitive evidence. Regarding prognosis, the cases in the reports by Sant'Anna et al. (2) and Simsek et al. (3), and the current case described long illness durations without significant progression, whereas the case reported by Ma et al. (1) had a shorter duration of illness and ended fatally. This suggests that the prognosis of TO in children may depend on the severity of airway stenosis. However, whether the severity of airway stenosis correlates with the etiology of TO requires further study.

In adults, the clinical symptoms of TO are non-specific and largely depend on the degree of tracheal and bronchial obstruction. Common presentations include chronic cough and fever, although some patients may remain asymptomatic (6). Pulmonary function tests are often normal, but as TO progresses, obstructive or restrictive ventilatory dysfunction may develop. Chest x-ray scans lack specific imaging features; however, secondary changes, such as pneumonia or atelectasis, may provide clues. Typical TO lesions on chest imaging may show increased bronchial wall density and irregular luminal changes.

Chest CT is a crucial diagnostic tool, revealing submucosal calcified nodules protruding into the tracheal and bronchial lumen, often described as “paving stone,” “undulating,” or “horseshoe-shaped” calcifications. Lesions predominantly affect the anterior and lateral walls of the trachea and bronchi, sparing the posterior wall. Bronchoscopy remains the gold standard for diagnosing TO. It reveals solitary or multiple nodules protruding into the tracheobronchial lumen, with a “cobblestone” or “stalactite cave-like” appearance. Pathological examination confirms TO, showing submucosal metaplastic cartilage or bone covered by normal or metaplastic epithelium. TO in children shares many clinical, pulmonary function, imaging, bronchoscopy, and pathological features with adults, but additional symptoms such as weak crying or cyanosis may occur depending on the age of onset. In the present case, the initial chest CT scan showed bilateral bronchial wall thickening without typical TO imaging features. A retrospective review after the definitive diagnosis identified scattered wall-adjacent nodules in the trachea and bilateral bronchi. Although chest CT scans in this case revealed predominant lesions in the right lung, bronchoscopic findings demonstrated more prominent nodular changes in the left bronchial tree. This discrepancy suggests that chest CT may have limitations in detecting early or subtle airway wall abnormalities in pediatric TO. In contrast, bronchoscopy plays a critical role in the early diagnosis of tracheobronchopathia osteochondroplastica in children by allowing direct visualization of characteristic endobronchial nodules.

The necessity of a bronchoscopic biopsy for TO is controversial. Some scholars argue that due to TO's benign nature and the typical bronchoscopic manifestations, pathological confirmation is unnecessary. Others highlight its importance in differential diagnosis (7, 8). In this case, the first biopsy pathology report indicated “mucosal hyperkeratosis,” prompting further investigation. A second biopsy confirmed the diagnosis. Sant'Anna et al. (2) also emphasized the value of pathology in ruling out other diseases. Therefore, bronchoscopic biopsy should be emphasized in suspected pediatric TO cases to avoid missed or incorrect diagnoses.

However, bronchoscopic biopsy in pediatric TO is challenging. In this case, the first biopsy failed to detect bone tissue, while the second biopsy led to bleeding, and the third biopsy specimen was inadequate. Similar challenges have been reported by Sant'Anna et al. (2) and others and have been attributed to superficial biopsy locations and the nature of pediatric TO tissue (9). Despite these difficulties, biopsy remains critical for definitive diagnosis and differential diagnosis. Patience and thoroughness are essential in completing these examinations.

TO in adults has no specific means of treatment and is mainly symptomatic. Severe tracheobronchial stenosis or airway obstruction can be treated with bronchoscopic laser ablation, balloon dilation, stent placement, or surgery (10, 11). Ma et al. (1) reported a pediatric case requiring interventional treatment for tracheal stenosis. Other cases, including those reported by Sant'Anna et al. (2), Simsek et al. (3), and in this paper, responded to symptomatic treatment, such as anti-infective therapy, with symptom improvement. Therefore, the treatment for children with TO is generally the same as that for adults. In addition, bronchoscopy in pediatric TO combined with pneumonia not only promotes the resolution of lung inflammation through aspiration but also slows TO progression and allows for a dynamic assessment of the disease.

## Data Availability

The original contributions presented in the study are included in the article/Supplementary Material, further inquiries can be directed to the corresponding author/s.
